# Approximation of EVLWI in severe COVID-19 pneumonia using quantitative imaging techniques: an observational study

**DOI:** 10.1186/s40635-025-00752-w

**Published:** 2025-05-19

**Authors:** Jonas Biehler, Marie Brei, Nina Pischke, Sebastian Rasch, Miriam Dibos, Johanna Erber, Roland M. Schmid, Rickmer F. Braren, Markus R. Makowski, Karl-Robert Wichmann, Kei Wieland Mueller, Wolfgang A. Wall, Tobias Lahmer

**Affiliations:** 1 Institute for Computational Mechanics, Technical University Munich, Boltzmannstr. 15, 85748 Munich, Germany; 2 Ebenbuild GmbH, Holzstraße. 28, 80469 Munich, Germany; 3 Medizinische Klinik und Poliklinik II, Klinikum Rechts der Isar, Technical University Munich, Ismaninger Straße 22, 81675 Munich, Germany; 4 Institute for Diagnostic and Interventional Radiology, Klinikum Rechts der Isar, Technical University Munich Ismaninger, Straße 22, 81675 Munich, Germany

**Keywords:** Extravascular lung water, Quantitative image analysis, ARDS, Pulmonary edema

## Abstract

**Background:**

This study aimed to approximate the level of extravascular lung water (EVLW) in patients with severe COVID-19 pneumonia using quantitative imaging techniques. The elevation of EVLW is known to correlate with the degree of diffuse alveolar damage and linked with the mortality of critically ill patients. Transpulmonary thermodilution (TPTD) is the gold standard technique to estimate the total amount of EVLW, but it is invasive and requires specialized equipment and trained personnel.

**Methods:**

The study included patients with severe COVID-19 who required chest CT scanning within the first 48 h of Intensive Care Unit (ICU) admission and had TPTD monitoring. Using in-house software tools for automatic semantic segmentation, lung masks were obtained for estimating the EVLW content. The results were compared with the TPTD measurements.

**Results:**

The results demonstrate a significant correlation between EVLW-TPTP measured by thermodilution and EVLW-CT estimated from the patient’s CT-image (*r* = 0.629, *p* = 0.0014).

**Conclusion:**

The study showed that quantitative imaging techniques using chest CT-scans could be used as a convenient and low-cost option for ICUs without TPTD equipment for the assessment of EVLW in severe COVID-19 pneumonia.

**Supplementary Information:**

The online version contains supplementary material available at 10.1186/s40635-025-00752-w.

## Background

The extravascular lung water (EVLW) is a parameter which represents the fluid outside the pulmonary vasculature. The level of EVLW does not only correlate with the degree of diffuse alveolar damage, but it is also linked with mortality of critically ill patients [3, 11].

The elevation of EVLW is observed in several critical disease constellations, e.g., in severe sepsis but foremost in patients with acute respiratory distress syndrome (ARDS) [11]. One form of ARDS with massively elevated EVLW levels is severe COVID-19 pneumonia.

With the onset of COVID-19 associated ARDS a cascade of an uncontrolled pulmonary inflammation with fluid accumulation, and potentially progression to fibrosis can be observed.

Although transpulmonary thermodilution (TPTD) is the gold standard technique to estimate the total amount of EVLW, it is an invasive, catheter-based technique, potentially associated with complications. In addition, TPTD requires equipment and trained personnel that is only available in specialized centers. An assessment of EVLW by means of automated quantitative image analysis would be a convenient and low-cost option for Intensive Care Units (ICUs) without the ability for TPTD measurements. As a step in this direction, the aim of this study is to evaluate the approximation of EVLW in severe COVID-19 pneumonia using quantitative imaging techniques. While previous studies have investigated the use of quantitative evaluations of CT-scans to estimate pulmonary fluid status with varying accuracy, the studies either focused on sheep [6], used non-routine CT-imaging techniques [19], or the studied patient cohort did not comprise patients with severe respiratory failure as primary condition [16].

## Methods

### Study design

Patients with severe COVID-19, who were treated in the medical ICU of the University hospital Klinikum rechts der Isar of the Technical University Munich, with need for CT scanning of the chest within the first 48 h after ICU admission as well as monitoring with TPTD for clinical reasons unrelated to the study were eligible for study inclusion.

TPTD measurements were performed 1–3 h before the CT scan.

This retrospective, observational study was approved (Approval No. 178/20S) by the Ethics Committee of the University hospital Klinikum rechts der Isar and written informed consent was obtained from all patients or their legal representatives.

Due to the retrospective and observational nature of this study, no formal sample size calculation was performed.

### Transpulmonary thermodilution measurements

For bedside measurement of the EVLW through TPTD (EVLW-TPTD) we used the PiCCO system (Pulsion Medical Systems SE, Feldkirchen, Germany) as described in [14, 15] with a 5-French thermistor-tipped catheter (Pulsiocath PV2015L20; Pulsion Medical Systems SE) placed in the abdominal aorta via the femoral artery. TPTD variables were calculated based on the analysis of the thermodilution curve after injection of 15 ml of iced 0.9% saline via a central venous catheter. The injection of the thermal indicator was performed in triplicate and each TPTD value represents the mean of three consecutive measurements. TPTD measurements were performed between 1 and 3 h before the CT scan. In addition to the measured EVLW, the indexed version (EVLWI) was computed using the predicted body weight according to the ARDS-Net formula [17].

### Quantitative image analysis

**Fig. 1:**
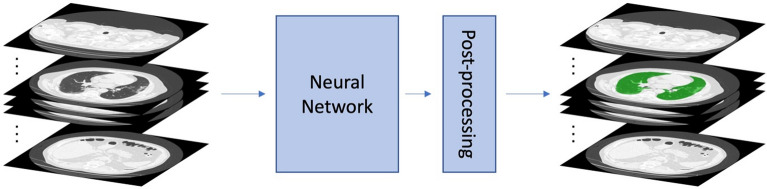
Illustration of overall image segmentation process using deep learning techniques

The chest CT-images were processed using in-house software tools for automatic semantic segmentation using deep learning techniques described in [5, 9, 12] and as illustrated in Fig. 1. Prior to running inference with the neural network, the CT-images were scaled down to an in-plane resolution of 256 × 256 to reduce computational complexity—mainly during the of training the model—and standardize input dimensions for the neural network. To restore the spatial resolution for the quantitative image analysis, the obtained lung masks were subsequently up-sampled to the original resolution of the CT-images and morphological closing was applied as a post-processing step. The resulting lung masks were then manually checked and corrected by an experienced operator, to ensure a consistent, high-quality lung segmentation for each patient. Using the lung masks we estimated the extravascular lung water content as follows: according to Protti et al. [10], we first computed the overall volume of the lung by:

$${V}_{\text{lung}}=\text{voxel volume}*N{V}_{lung\, mask},$$

where $N{V}_{lung\, mask}$ is the number of voxels in the lung mask. Next, the per voxel water volume was computed by

$$\text{voxel water }volume=\left[1-\frac{voxel\, density}{-1000}\right]*\text{voxel volume},$$

where the voxel density is expressed in Hounsfield Units (HU). The formula above essentially conflates water and tissue content within a voxel as an approximation of the water content of a voxel. The overall lung water content was then computed by summing up the per voxel quantities and used as an estimate for the extra-vascular lung water, hereafter referred to as EVLW-CT. Note that this estimate includes both extravascular and vascular components as those are difficult to differentiate from CT alone, especially in the context of severe pathologies and lower resolution CT scan. To address this limitation, we incorporated a refined estimate of EVLW, referred to as EVLW-CT-Corr. This estimate was calculated by subtracting a standardized pulmonary blood volume (PBV) value of 418 ml from the total lung water content for all study participants. The value of 418 ml represents the mean PBV reported by Hermann et al. [4] in a large cohort study involving 727 participants.

However, it should be noted that, while this adjustment provides a more specific approximation of extravascular lung water it may also introduce an additional bias due to inter-individual variations in vascular anatomy and perfusion [1, 18]. To ensure consistency in the analysis, we have therefore included both quantities.

Both estimates can furthermore be indexed using the predicted body weight according to the ARDS-Net formula [17]. The respective quantities are referred to as EVLWI-CT and EVLWI-CT-Corr from here on. In addition, we decided to also compute the quantities used in Saugel et al. [16] to enable a better comparison of results. More specifically, we computed an approximation to the tissue volume (TV) as described in Saugel et al. [16] using

$$\begin{aligned}TV&=(volume\, of\, well\, aerated\, lung\, tissue*0.3)\\  &\quad+ (volume\, of\, poorly\, aerated\, lung\, tissue*0.7)\\ &\quad+(volume\, of\, non-aerated\, lung\, tissue*1.0),\end{aligned}$$

with the same HU-thresholds to discern between well-aerated (− 900 to − 500 HU), poorly aerated (− 499 to − 100 HU), non-aerated (− 99 to + 100 HU) and hyperinflated (< − 900 HU) tissue. We also computed an indexed version (TVI) based on TV and the predicted bodyweight as in the case of EVLWI. In addition, we calculated the mean weighted index of voxel aqueous density (VMWaq) according to Saugel et al. using

$$\begin{aligned}VMWaq &= ( ( {{\text{ number }}( {\text{n}} ){\text{of well}} - {\text{aerated lung tissue voxels }}*0.3} ) \\ &+ ( {{\text{n of poorly aerated tissue voxels }}*0.7} ) \\ &+( {{\text{n of non}} - {\text{aerated tissue voxels }}*1.0} ) )/\\&{{\text{total number of voxels in lung mask}}}.\end{aligned}$$

### Statistical analysis

We computed the Spearman rank-correlation to analyze the relationship between measured EVLW-TPTD and EVLW-CT, TV, as well as VMWaq estimated from the patient’s CT-image. We used the Spearman rank-correlation as it does not assume a linear relationship and is robust to potential variability introduced by the inclusion of vessel volume in the total lung water volume derived from the CT. The same analysis was conducted for the indexed versions EVLWI-TPTD, EVLWI-CT, and TVI, respectively. Furthermore, Bland–Altman plots were used to compare TPTD-based and CT-based estimations of EVLW and identify systematic differences between the two approaches. In this context, we also looked at the percentage error (PE) which is calculated as follows:

$$PE \left(\%\right)=100\%*1.96*\frac{{\sigma }_{TPDT-CT}}{{\mu }_{TPDT}},$$

where ${\sigma }_{TPTD-CT}$ is the standard deviation of the differences of measurements from CT and TPTD, and ${\mu }_{TPTD}$ is the mean of measurements from the reference method, i.e., the TPTD [2]. Statistical tests were conducted in an exploratory manner on a two-sided 5% significance level. Descriptive data are presented as absolute and relative frequencies (categorical data) or as median and 25th and 75th percentile (continuous data). All statistical analyses and data processing were performed using Python version 3.9, utilizing NumPy, pandas, statsmodels and SciPy.

## Results

### Patients

In total, 25 critically ill patients with severe COVID-19 pneumonia on mechanical ventilation were included in this observational study. Two patients were excluded as the height was not documented and hence the predicted body weight could not be computed. Thus, we included 23 patients in the final analysis. The patients’ characteristics are presented in Table 1.

**Table 1:** Patient characteristics

	All (*n* = 23)
Sex (female/male)	5/18
Age (years)	70 (58.5–75.5)
Height (cm)	175 (170–180)
Predicted body weight (kg)	70,7 (63,87–5,28)
Body mass index (kg/m^2^)	26.2 (22.9–27.8)
Time between intubation and CT scan (hours)	4.5 (2.5–6.5)
PaO_2_/FiO_2_ (mmHg)	70 (40–105)
Fluid balance within first 24 h (liters)	1.8 (1.2–3.5)
Need for norepinephrine (%)	100
Need for dialyses within first 24 h (%)	15

Data are presented as counts, percentages or median with 25th percentile–75th percentile

### Pulmonary fluid status assessed through quantitative imaging

Descriptive statistics from quantitative CT analysis including information on the distribution of hyperinflated, well-, poorly, and non-aerated lung tissue are shown in Table 2 along with the computed values for EVLW-CT, EVWL-CT-Corr, TV, VMWaq, and the indexed variants EVLWI-CT, EVLWI-CT-Corr and TVI. Median EVLW-CT was 1.66 (1.27–2.08) ml, median EVLWI-CT was 23.08 (19.25–28.22), median EVLW-CT-Corr was 1.18 (0.79–1.59), median EVLWI-CT-Corr was 16.69 (11.86–22.01), median TV was 1.49 (1.25–1.96), median TVI was 21.36 (19.17–27.8), and median VMWaq was 0.42 (0.34–0.45). The quantitative CT analysis for each individual patient is provided in the supplementary materials (Appendix A).

**Table 2:** Population statistics (N = 23) from transpulmonary thermodilution measurements and quantitative CT analysis

Variable	Mean ± SD	Median (Q1-Q3)	Min	Max
EVLW-TPTD [l]	1.14 ± 0.3	1.1 (0.94–1.32)	0.58	1.81
EVLWI-TPTD [ml/kg]	16.48 ± 3.79	16.0 (14.0–19.5)	11.0	24.0
EVLW-CT [l]	1.65 ± 0.48	1.66 (1.27–2.08)	0.99	2.5
EVLWI-CT [ml/kg]	24.09 ± 7.34	23.08 (19.25–28.22)	13.14	43.49
EVLW-CT-Corr [l]	1.17 ± 0.48	1.18 (0.79–1.59)	0.51	2.01
EVLWI-CT-Corr [ml/kg]	17.05 ± 7.13	16.69 (11.86–22.01)	6.75	35.06
TV [l]	1.6 ± 0.45	1.49 (1.25–1.96)	0.93	2.48
TVI [ml/kg]	23.37 ± 6.83	21.36 (19.17–27.8)	12.41	41.63
VMWaq	0.41 ± 0.08	0.42 (0.34–0.45)	0.28	0.55
Total lung volume [l]	3.99 ± 0.98	3.96 (3.37–4.45)	1.92	5.72
Volume hyperinflated [l]	0.32 ± 0.28	0.22 (0.1–0.5)	0.03	1.01
Percentage Hyperinflated [%]	7.33 ± 5.76	5.7 (2.36–10.86)	0.78	18.07
Volume well-aerated [l]	2.28 ± 0.71	2.38 (1.88–2.71)	0.72	3.61
Percentage well-aerated [%]	56.98 ± 11.15	57.65 (45.52–66.05)	37.71	74.11
Volume poorly aerated [l]	0.89 ± 0.42	0.86 (0.58–1.17)	0.27	1.62
Percentage poorly aerated [%]	22.81 ± 10.43	22.18 (14.46–28.02)	6.33	43.56
Volume non-aerated [l]	0.3 ± 0.23	0.28 (0.12–0.4)	0.07	1.02
Percentage non-aerated [%]	7.72 ± 5.46	8.14 (2.69–9.06)	1.71	24.16

*SD* standard deviation, Q1 25 quantile, *Q3 75* quantile

### Pulmonary fluid status assessed with transpulmonary thermodilution

Descriptive statistics on TPTD-derived EVLW-TPTD and EVLWI-TPTD for the whole patient cohort are shown in Table 2. Median EVLW-TPTD was 1.14 (0.94–1.32), respectively. Median EVLWI-TPTD was 16.48 (14.0–19.5) ml/kg. Data on TPTD measurements are shown individually for each patient the supplementary materials (Appendix A).

### Correlation analysis

**Fig. 2:**
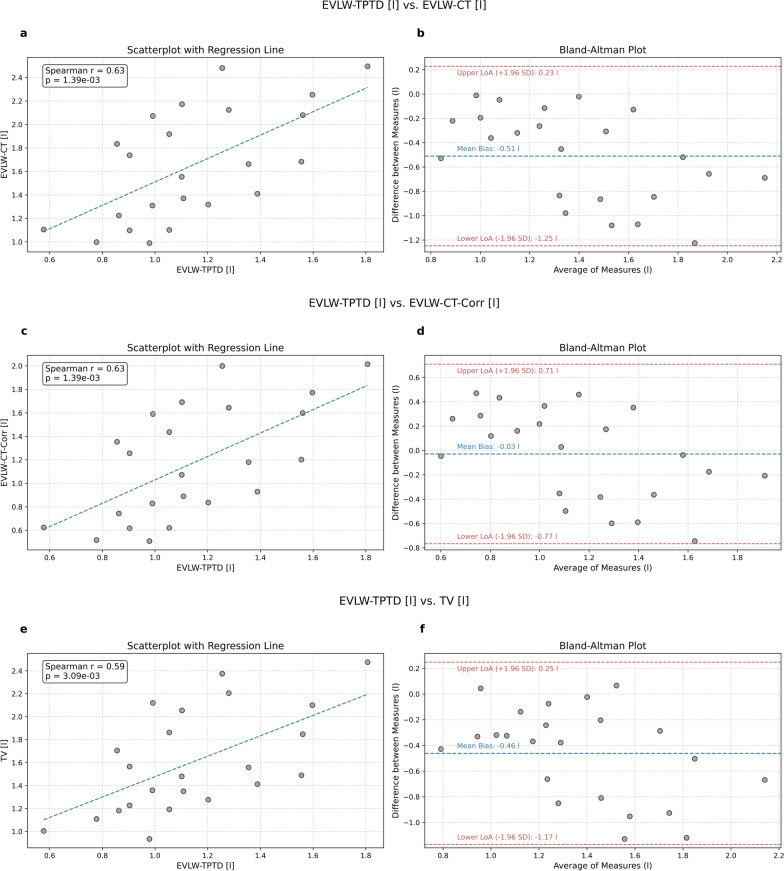
Relationship and agreement between extra-vascular lung water measured by thermodilution (EVLW-TPTD) and extra-vascular lung water (EVLW-CT, EVLW-CT-Corr) and tissue volume (TV) estimated from CT-images. The left panel presents scatterplots illustrating the relationship between **a** EVLW-TPTD and EVLW-CT, **b** EVLW-TPTD and EVLW-CT-Corr, and **c** EVLW-TPTD and TV with a regression line, Spearman correlation (*r*) and *p*-value. The right panel displays Bland–Altman plots assessing the agreement between **d** EVLW-TPTD and EVLW-CT, **e** EVLW-TPTD and EVLW-CT-Corr, and **f** EVLW-TPTD and TV, including the mean bias and 95% limits of agreement (LoA)

**Fig. 3:**
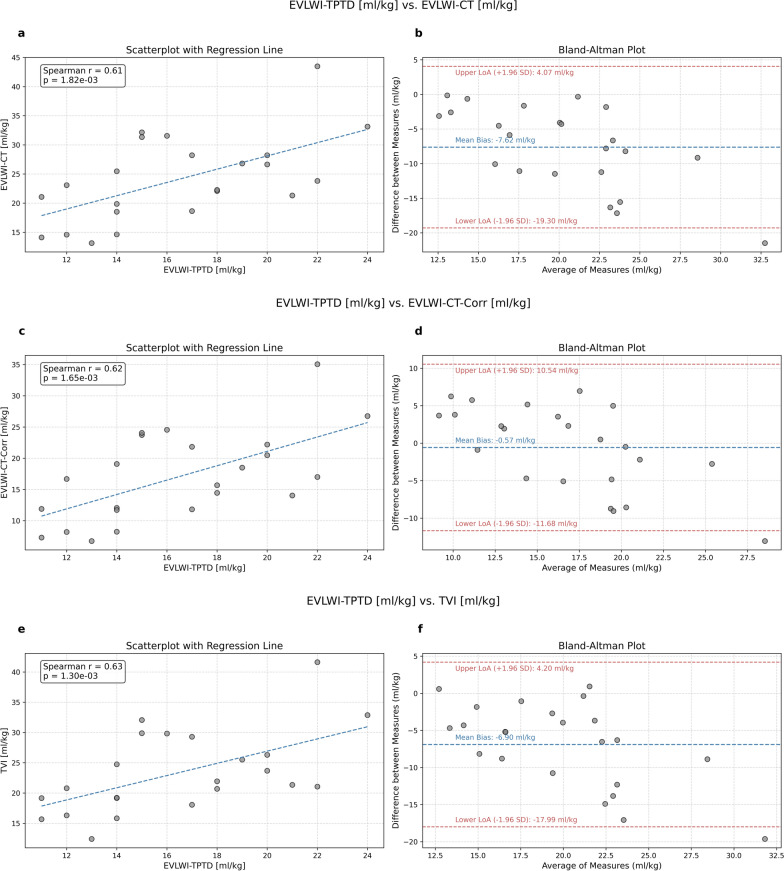
Relationship and agreement between extra-vascular lung water index measured by thermodilution (EVLWI-TPTD) and extra-vascular lung water index (EVLWI-CT, EVLWI-CT-Corr) and tissue volume index (TVI) estimated from CT-images. The left panel presents scatterplots illustrating the relationship between **a** EVLWI-TPTD and EVLWI-CT, **b** EVLWI-TPTD and EVLWI-CT-Corr, and **c** EVLWI-TPTD and TVI with a regression line, Spearman correlation (r) and *p*-value. The right panel displays Bland–Altman plots assessing the agreement between **d** EVLWI-TPTD and EVLWI-CT, **e** EVLWI-TPTD and EVLWI-CT-Corr, and **f** EVLWI-TPTD and TVI, including the mean bias and 95% limits of agreement (LoA)

**Fig. 4:**
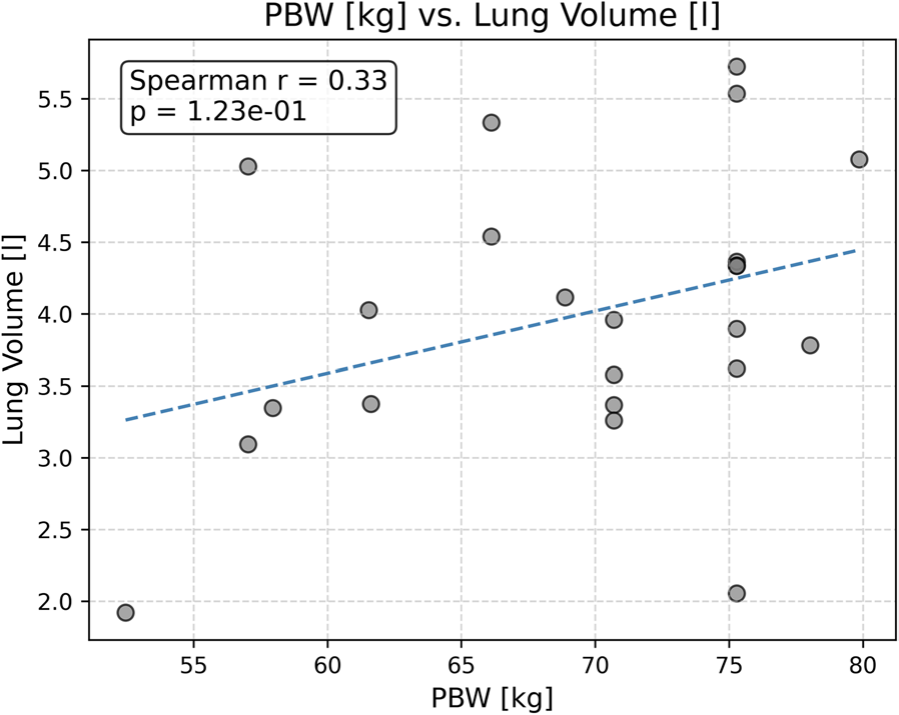
Scatterplot showing the relationship between the lung volume computed from CT and predicted body weight (PBW) with a regression line, Spearman correlation (*r*) and *p*-value

There was a significant correlation between EVLW-TPTP measured by thermodilution and EVLW-CT(-Corr) estimated from the patient’s CT-image (*r* = 0.629, *p* = 0.0014) as well as between EVLW-TPTD and TV (*r* = 0.589, *p* = 0.0031). The corresponding scatter plots are shown in Fig. 2a, c and e. A similar correlation was found for the indexed versions of these quantities, i.e., between EVLWI-TPTD and EVLWI-CT(-Corr) (*r* = 0.614, *p* = 0.0018) as well as between EVLWI-TPTD and TVI (*r* = 0.629, *p* = 0.0013). The corresponding data are shown in Fig. 3a, c, e, respectively. We did, however, not find a significant correlation between VMWaq and EVLWI-TPTD (r = 0.177, p = 0.4201), data shown in the supplementary Figure S1. We have also investigated the relationship between lung volume estimated from predicted body weight and lung volume computed from the quantitative image analysis, data shown in Fig. 4. Interestingly, we did not find a significant correlation (r = 0.331, p = 0.123) between predicted body weight and lung volume.

### Bland–Altman analysis

We conducted a Bland–Altman analysis to assess the agreement between TPTD- and CT-derived quantities. For the comparison of EVLW-TPTD and EVLW-CT the analysis revealed an absolute bias of −0.51 l with limits of agreement (LoA) ranging from − 1.23 l to 0.21 l and a PE of 64.6% (Fig. 2b). For the corrected estimate (EVLW-CT-Corr), the comparison with EVLW-TPTD showed a bias of 0.03 l with lower and upper LoA at −0.77 l and 0.71 l and a PE of 64.6% (Fig. 2d). Comparing EVWL-TPTD with the CT-derived TV (Fig. 2f) indicates a bias of − 0.46 l with lower LoA of − 1.16 l, upper LoA of 0.23 l and a PE or 62.2%. For the indexed quantities, the analysis showed a bias of −7.62 ml/kg with lower LoA of −19.04 ml/kg, upper LoA of 3.81 ml/kg and a PE of 70.9% for the comparison of EVLWI-TPTD with EVLWI-CT (Fig. 3b), a bias of − 0.57 ml/kg, upper and lower LoA of 10.54 ml/kg and − 11.68 ml/kg and a PE of 67.4% for the comparison of EVLWI-TPTD with EVLWI-CT-Corr (Fig. 3d) and a bias of − 6.90 ml/kg with lower and upper LoA of − 17.75 ml/kg and 3.95 ml/kg and a PE of 67.3% for the comparison of EVLWI-TPTD with TVI (Fig. 3f).

## Discussion

To the best of our knowledge this is the first study that estimates EVLW using CT-based quantitative imaging techniques in critically ill COVID-19 patients. We found a robust correlation between EVLW estimated from CT-scans and measured TPTD values. The correlation coefficient between EVLWI-TPTD and EVLWI-CT is in agreement with results obtained from patients with non-COVID-19 related ARDS [19]. However, the correlation reported by Zhang et al. [19] was stronger, likely because the authors did not use routine CT-images but applied a dedicated, standardized imaging protocol with image acquisitions performed during an end-expiratory hold.

Interestingly, our correlation results differ markedly from the results reported by Saugel et al.[16] who did not find a strong correlation between EVLW as measured by TPTD and CT, respectively. The main reason might be the study cohort, e.g., primary (COVID-19 related) versus secondary ARDS (sepsis related) and the timepoint of imaging which was during the acute phase of COVID-19 ARDS in our cohort.

For the uncorrected estimates, the Bland–Altman analysis revealed a systematic overestimation of EVLW by the CT-based methods. Specifically, the bias for EVLW-CT in comparison to EVLW-TPTD was −0.51 l, with limits of agreement (LoA) ranging from − 1.23 l to 0.21 l. The observed overestimation of CT-derived quantities compared to TPTD measurements can be attributed to the fundamental differences in the methodologies as described in the methods section. However, the observed bias for EVLW-CT aligns closely with the mean pulmonary blood volume (PBV) of 481 ml reported by Hermann et al. [4]. Thus, correcting EVLW-CT with this value leads to an almost unbiased estimate (bias of 0.03 l and LoA ranging from − 0.77 l to 0.71 l for EVWL-CT-Corr). For the indexed quantities, EVLWI-CT-Corr also showed better agreement with EVLWI-TPTD, with a bias of −0.57 ml/kg (LoA: − 11.68 ml/kg to 10.54 ml/kg) compared to the uncorrected EVLWI-CT, which had a bias of −7.62 ml/kg (LoA: − 19.04 ml/kg to 3.81 ml/kg). These results highlight the benefit of correcting for pulmonary blood volume (PBV) in improving agreement. Nevertheless, the wide LoA and high percentage error (PE) across all comparisons (ranging from 62.2% to 70.9%) indicate substantial variability which likely result from both methods. Our current CT-derived estimates inherently include vascular components due to the lack of vessel segmentation, leading to a systematic overestimation of EVLW. Although the corrected estimates (EVLW-CT-Corr) subtract a standardized PBV to address this, inter-individual variations in vascular anatomy and perfusion remain unaccounted for, introducing potential variability across the dataset. The variability may be further amplified by the lack of standardized imaging protocols for the CT acquisition which can influence the observed water distribution and segmentation accuracy, leading to increased measurement uncertainty. In addition, TPTD itself is subject to multiple limitations that may contribute to the observed variability. Factors such as tidal volume, PEEP levels or the PaO_2_/FiO_2_ ratio can influence EVLW measurements with TPTD [7, 8]. Clinical conditions like pulmonary embolism, pleural effusion, or heterogeneous ARDS can further affect accuracy, as pulmonary blood flow redistribution in ARDS may underestimate EVLW, while large pleural effusions can lead to overestimation [8]. Moreover, the study of Sakka et al. [13] has shown that TPTD tends to underestimate actual values compared to the double-indicator dilution method, particularly in cases of high EVLWI (above 12 ml/kg) and overestimates it at low-normal values,. With a mean EVLWI of 24.09 ml/kg this effect might have a relevant influence on our study results.

In addition, the poor correlation between predicted body weight and lung volume is an interesting finding. The use of predicted body weight assumes that in connection with mechanical ventilation volutrauma can be minimized by adjusting the tidal volume according to the patient’s lung capacity. In the absence of quantitative imagine techniques, physicians hitherto rely on predicted body weight as a proxy to actual lung capacity. The results obtained in this study challenge the validity of this assumption. However, since the CT-scans evaluated in this study were not obtained at defined pressure levels, further investigations with a larger patient cohort are necessary to analyze the relation between predicted body weight and lung capacity more rigorously.

### Sensitivity of VMWaq

Aside from our data not showing a significant correlation between VMWaq and EVLW-TPTD, VMWaq is, in our opinion, not well suited to assess extravascular lung water. VMWaq is quite sensitive to the fraction of hyperinflated lung tissue which is not included in the numerator of the formula to compute VMWaq. Consequently, VMWaq will change considerably if the fraction of hyperinflated lung tissue changes, which happens, e.g., throughout the course of the ventilation cycle. In addition, different ventilator settings such as increased pressure levels like a higher positive end-expiratory pressure (PEEP) or driving pressure will have a more pronounced effect on VMWaq as compared to say EVLW-CT.

### Clinical implications

As the CT-scans were performed under routine clinical conditions the algorithm will likely generalize under various clinical constellations. Because EVLW parameters are closely related to higher morbidity and mortality rates our findings may be of general importance in the workup of ICU patients.

While the TPTD measurements could be used as continuous EVLW measurements over several days to potentially monitor ARDS treatment, the CT-based EVLW estimation is correlated to a specific timepoint. However, using a CT scan-based estimation also allows the correlation to the lung structure and pathological findings that are not visible using TPTD alone. This capability is particularly relevant in conditions such as ARDS, where regional heterogeneity in lung pathology is common.

The observed variability between CT- and TPTD-derived measurements limits the direct applicability in clinical routine at this stage and highlights the importance of context when interpreting EVLW values, particularly in critically ill patients with complex lung pathologies.

## Limitations

One limitation of the current study is the absence of a standardized imaging protocol during CT acquisition. Furthermore, the respiration state at CT was not available as the data were collected retrospectively. These factors can influence the observed water distribution and segmentation accuracy and therefore increase measurement uncertainty. The results of the quantitative image analysis might be more consistent if the image were always acquired at an expiratory or inspiratory hold. Another limitation is that the blood vessels within the lungs were not explicitly accounted for in the image analysis. The computed EVLW-CT represents the total water content within the segmented lung area, including both vascular and extravascular components, which leads to a systematic overestimation of EVLW from the CT analysis. Partly, this is due to the lack of a robust automatic vessel segmentation approach for these severely damaged lungs. To partially address this, we introduced a refined estimate, EVLW-CT-Corr, calculated by subtracting a standardized pulmonary blood volume (PBV) of 418 ml from the total lung water content. While this adjustment leads to an unbiased estimation of EVLW, it should be noted that individual variations in vascular anatomy and perfusion are not fully captured. Future studies integrating complementary imaging modalities, such as dual-energy CT, or more advanced segmentation techniques for the pulmonary vasculature may help to quantify the PBV more precisely and patient-specific. Since the data for this study were collected retrospectively, we were unable to precisely control the time delay between image acquisition and TPTD measurement, which also could affect the accuracy of the results. We plan to address this issue in future work by conducting TPTD measurements within close proximity to the CT scan. Furthermore, the relatively homogeneous patient population in this study is a limitation, as it may restrict the generalizability of our findings to other pathologies or more diverse clinical settings. Future studies should aim to validate these results in larger and more heterogeneous cohorts to enhance their clinical applicability. Lastly, as this work is limited to data from a single center, the study should be repeated with a larger cohort from multiple centers to further validate the results obtained in this study.

## Conclusion

In contrast to previous studies [16], we found a correlation between the pulmonary fluid status assessed with TPTD and quantitative imaging techniques in severe ARDS. We found that the correlation was slightly more pronounced for the un-indexed version EVLW-TPTD and EVLW-CT(-Corr) (*r* = 0.626) as compared to the indexed version of the EVLWI-TPTD and EVLWI-CT(-Corr) (*r* = 0.614). While our quantitative analysis still required manual fine-tuning of the segmentation in particular with regard to the existing pleural effusions, the future version of our software should allow for fully automatic lung segmentation and hence estimation of extravascular lung water based on the quantities described in this paper.

The Bland–Altman analysis, however, revealed significant variability between CT-derived and TPTD-derived measurements, as indicated by the high percentage errors (62.2–70.9%) and relatively wide limits of agreement. These findings highlight the challenges of achieving accurate and precise results with the current CT-based method, particularly in the absence of a segmentation of the pulmonary vessels and standardized imaging protocols, both of which can be addressed in future work.

Despite the current limitations, these methods could become a valuable tool in centers without the capability to measure EVLWI through a transpulmonary thermodilution device. However, further studies in larger and more diverse populations, combined with enhanced imaging techniques, are necessary to validate the clinical utility of this approach and address the observed variability.

## Supplementary Information

Supplementary Material 1. Figure S1. Extra vascular lung water index measured by thermodilutionplotted against extra VMWaq estimated from CT-images with a regression line, Spearman correlationand *p*-value.

## Data Availability

The datasets generated during the current study are available from the corresponding author on reasonable request.

## Appendix

See Table 3.

**Table 3:** Data from transpulmonary thermodilution measurements and quantitative CT analysis

ID	TPTD		CT															
	EVLW	EVLWI	EVLW	EVLWI	EVLW-Corr	EVLWI-Corr	TV	TVI	VMWaq	Total Lung	V_Hyperinflated_		V_Well-Aerated_		V_Poorly Aerated_		V_Non-Aerated_	
	[l]	[ml/kg]	[l]	[ml/kg]	[l]	[ml/kg]	[l]	[ml/kg]		Volume [l]								
											[l]	[%]	[l]	[%]	[l]	[%]	[l]	[%]
1	1,597	20,0	2,253	28,215	1,7722	22,192	2,101	26,306	0,414	5,078	0,571	11,247	2,375	46,78	1,417	27,907	0,396	7,806
2	1,807	24,0	2,495	33,147	2,0143	26,758	2,475	32,879	0,432	5,724	1,013	17,689	2,43	42,449	1,044	18,245	1,015	17,733
3	1,054	14,0	1,918	25,477	1,4369	19,088	1,863	24,749	0,427	4,365	0,201	4,61	2,479	56,791	1,002	22,957	0,418	9,575
4	1,101	19,0	1,554	26,805	1,0726	18,506	1,48	25,533	0,442	3,347	0,055	1,653	1,929	57,648	0,893	26,692	0,276	8,237
5	1,28	17,0	2,125	28,223	1,6437	21,834	2,206	29,3	0,398	5,537	0,433	7,824	3,614	65,274	0,959	17,312	0,45	8,135
6	0,856	15,0	1,834	32,16	1,3534	23,727	1,705	29,899	0,551	3,095	0,033	1,073	1,342	43,355	0,793	25,619	0,748	24,164
7	0,992	15,0	2,072	31,334	1,5908	24,059	2,12	32,07	0,398	5,334	0,365	6,841	3,157	59,189	1,5	28,132	0,123	2,307
8	0,863	14,0	1,224	19,867	0,7432	12,061	1,182	19,181	0,35	3,376	0,105	3,106	2,502	74,114	0,401	11,865	0,151	4,474
9	1,561	20,0	2,079	26,647	1,5983	20,483	1,848	23,68	0,489	3,782	0,03	0,784	1,664	43,985	1,471	38,885	0,319	8,441
10	0,903	12,0	1,737	23,08	1,2564	16,69	1,566	20,799	0,432	3,622	0,13	3,579	2,021	55,788	0,936	25,836	0,305	8,41
11	0,903	12,0	1,098	14,586	0,617	8,1965	1,228	16,309	0,283	4,338	0,655	15,109	3,159	72,817	0,275	6,333	0,088	2,024
12	1,102	16,0	2,173	31,548	1,6917	24,563	2,054	29,831	0,499	4,116	0,103	2,508	1,822	44,269	1,59	38,624	0,395	9,602
13	1,389	21,0	1,41	21,319	0,9286	14,044	1,412	21,361	0,311	4,54	0,663	14,61	2,97	65,425	0,607	13,366	0,097	2,127
14	1,202	17,0	1,317	18,631	0,8362	11,828	1,277	18,064	0,323	3,96	0,415	10,471	2,64	66,678	0,549	13,855	0,101	2,552
15	0,979	13,0	0,989	13,139	0,5081	6,7492	0,935	12,415	0,455	2,055	0,031	1,519	1,217	59,209	0,386	18,799	0,299	14,551
16	0,577	11,0	1,105	21,063	0,624	11,894	1,005	19,16	0,523	1,921	0,037	1,939	0,724	37,712	0,837	43,557	0,202	10,533
17	0,778	11,0	0,998	14,112	0,5168	7,3091	1,109	15,685	0,31	3,577	0,606	16,951	2,325	64,99	0,459	12,838	0,09	2,517
18	1,054	14,0	1,101	14,63	0,6203	8,2405	1,192	15,839	0,275	4,335	0,783	18,072	2,982	68,781	0,32	7,375	0,074	1,708
19	1,555	22,0	1,683	23,809	1,2023	17,006	1,49	21,07	0,442	3,369	0,259	7,682	1,326	39,373	1,286	38,189	0,191	5,676
20	1,355	18,0	1,662	22,08	1,1812	15,69	1,558	20,696	0,4	3,896	0,222	5,702	2,223	57,061	0,864	22,177	0,286	7,343
21	1,108	18,0	1,371	22,276	0,8899	14,46	1,35	21,945	0,335	4,028	0,344	8,545	2,704	67,148	0,607	15,069	0,114	2,838
22	1,255	22,0	2,481	43,488	1,9995	35,055	2,374	41,628	0,472	5,029	0,111	2,205	2,708	53,853	1,618	32,177	0,429	8,538
23	0,99	14,0	1,31	18,523	0,8285	11,719	1,359	19,215	0,417	3,26	0,159	4,874	2,211	67,816	0,613	18,802	0,266	8,166

*TPTD* transpulmonary thermodilution, *CT* computed tomography, *ELVW* extravascular lung water, *EVLWI* extravascular lung water index, *TV* tissue volume, *TVI* tissue volume index, *VMW*_*aq*_ mean weighted index of voxel aqueous density, *V* volume

## Abbreviations

EVLW: Extra vascular lung water
EVLWI: Extra vascular lung water index
ICU: Intensive Care Unit
CT: Computed tomography
PEEP: Positive end-expiratory pressure
TPTD: Transpulmonary thermo dilution
