# Supplementary figures and images for: Approximation of EVLWI in severe COVID-19 pneumonia using quantitative imaging techniques: an observational study
Source: Intensive Care Med Exp. 2025 May 19;13:52. doi: 10.1186/s40635-025-00752-w (PMC12089548; doi:10.1186/s40635-025-00752-w)

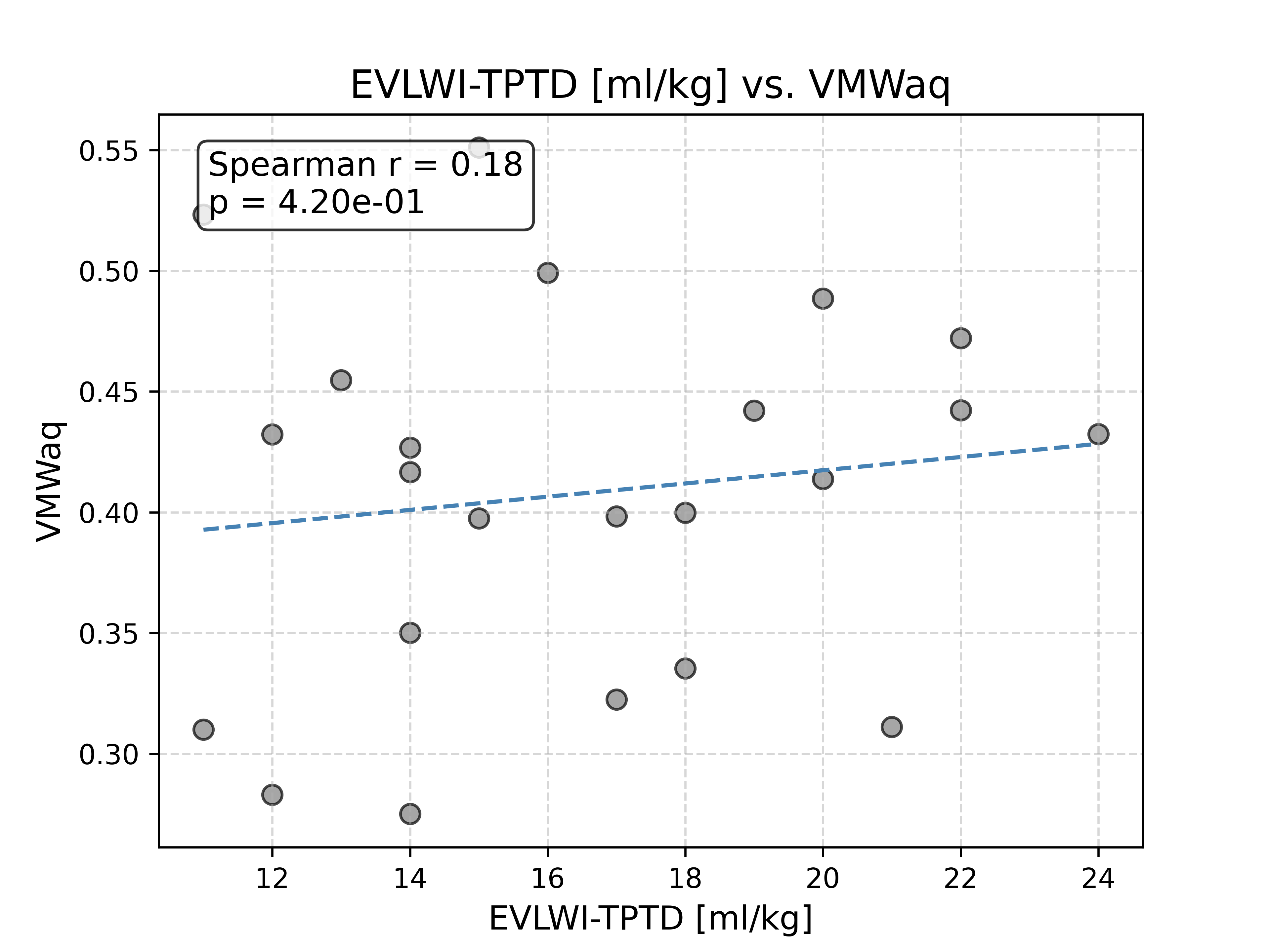

Supplement: Supplementary file 1 — Supplementary Material 1. Figure S1. Extra vascular lung water index measured by thermodilutionplotted against extra VMWaq estimated from CT-images with a regression line, Spearman correlationand p-value. [file 40635_2025_752_MOESM1_ESM.png]
